# Identification of a Novel Aminopeptidase P-Like Gene (*OnAPP*) Possibly Involved in Bt Toxicity and Resistance in a Major Corn Pest (*Ostrinia nubilalis*)

**DOI:** 10.1371/journal.pone.0023983

**Published:** 2011-08-24

**Authors:** Chitvan Khajuria, Lawrent L. Buschman, Ming-Shun Chen, Blair D. Siegfried, Kun Yan Zhu

**Affiliations:** 1 Department of Entomology, Kansas State University, Manhattan, Kansas, United States of America; 2 USDA-ARS, Hard Winter Wheat Genetics Research Unit, Kansas State University, Manhattan, Kansas, United States of America; 3 Department of Entomology, University of Nebraska, Lincoln, Nebraska, United States of America; New England Biolabs, Inc., United States of America

## Abstract

Studies to understand the *Bacillus thuringiensis* (Bt) resistance mechanism in European corn borer (ECB, *Ostrinia nubilalis*) suggest that resistance may be due to changes in the midgut-specific Bt toxin receptor. In this study, we identified 10 aminopeptidase-like genes, which have previously been identified as putative Bt toxin receptors in other insects and examined their expression in relation to Cry1Ab toxicity and resistance. Expression analysis for the 10 aminopeptidase-like genes revealed that most of these genes were expressed predominantly in the larval midgut, but there was no difference in the expression of these genes in Cry1Ab resistant and susceptible strains. This suggested that altered expression of these genes was unlikely to be responsible for resistance in these ECB strains. However, we found that there were changes in two amino acid residues of the aminopeptidase-P like gene (*OnAPP*) involving Glu^305^ to Lys^305^ and Arg^307^ to Leu^307^ in the two Cry1Ab-resistant strains as compared with three Cry1Ab-susceptible strains. The mature OnAPP contains 682 amino acid residues and has a putative signal peptide at the N-terminus, a predicted glycosylphosphatidyl-inositol (GPI)-anchor signal at the C-terminal, three predicted *N*-glycosylation sites at residues N178, N278 and N417, and an *O*-glycosylation site at residue T653. We used a feeding based-RNA interference assay to examine the role of the *OnAPP* gene in Cry1Ab toxicity and resistance. Bioassays of Cry1Ab in larvae fed diet containing *OnAPP* dsRNA resulted in a 38% reduction in the transcript level of *OnAPP* and a 25% reduction in the susceptibility to Cry1Ab as compared with larvae fed GFP dsRNA or water. These results strongly suggest that the *OnAPP* gene could be involved in binding the Cry1Ab toxin in the ECB larval midgut and that mutations in this gene may be associated with Bt resistance in these two ECB strains.

## Introduction

The insecticidal properties of the *Bacillus thuringiensis* (Bt) toxins have been extensively exploited for insect pest control. The spores and crystals of Bt have been used as biopesticides for almost 60 years in the areas of forestry, agriculture, and vector-born disease control [1], [2]. The importance of the Bt toxins in the management of the insect pests has increased dramatically with the development of transgenic plants that have been genetically modified to express Bt protein toxins in their tissues [3], [4]. However, there are concerns that wide-spread use of transgenic crops expressing Bt toxins may lead to the development of resistance among target pests and shorten the life of Bt technology. Therefore, identification of the genes involved in the Bt toxin interactions will be fundamental to developing effective resistance management strategies that will be useful in preventing or at least delaying the onset of resistance in insect populations.

The mode of action for Bt toxin in which the relatively inert crystalline protoxin form is changed into the cytotoxic form involves several steps [1]; however, there are two competing models that have been proposed to explain the mode of action of Bt toxins. In both models the initial steps are identical, including solubilization of protoxin, activation of the soluble protoxin by the gut proteases into a Cry monomeric toxin, and binding of the toxin to the cadherin receptor [5]. The pore formation model [6] suggests that cadherin causes toxin oligomerization and the oligomeric cry toxin then binds to GPI-anchored receptors which facilitates toxin insertion into the membrane and pore formation, which leads to osmotic imbalance, cell lysis, and eventually death of the target insects [1], [7]. In contrast, the signal transduction model [8] proposes that monomeric Cry1Ab binds to cadherin and initiates an Mg^+2^ –dependant signaling pathway that promotes cell death. In addition to cadherin, there are many other cry toxin receptors that have been reported such as GPI-aminopeptidase N, GPI-alkaline phosphatase, GPI-ADAM metalloprotease, glycolipids, glyco-conjugate, V-ATP synthase subunit, and actin [3], [9], [10], [11].

A number of insect species have developed resistance to Cry toxins when exposed to selection pressure under laboratory conditions [12]. Two known mechanisms of Bt resistance have been identified in insects, which include proteinase-mediated and receptor-mediated resistance [13]. However, the most common mechanism of Cry toxin resistance reported among resistant strains involves mutations that affect the assembly of cadherin receptor molecules [12]. The mutations in the cadherin gene have been shown to be genetically linked to Cry1A resistance in *Heliothis virescens*, *Pectinophora gossypiella,* and *Helicoverpa armigera* [14]–[16]. A mutation of an aminopeptidase N gene has also been associated with resistance to Cry1Ac in *Helicoverpa armigera* [17]. Moreover, in *Spodoptera litura*, reducing the expression of the aminopeptidase N gene with dsRNA resulted in reduced susceptibility to Cry1Ca toxin, suggesting that the aminopeptidase N gene was also involved in the toxicity [18].

The European corn borer (ECB, *Ostrinia nubilalis* Hübner) is one of the most damaging pests of corn in Europe and North America. Transgenic corn expressing Bt toxins has been very successful in managing the ECB. Resistance to Cry toxins in ECB has developed under laboratory selection conditions [19]–[22]. The resistance mechanism in the Dipel-resistant ECB has been linked to reduced proteases in the resistant strain [23], [24]. In another study, comparison of the midgut protease between Cry1Ab resistant and susceptible strains showed no consistent difference [25], which suggested that the resistance mechanism might involve modified midgut receptors [19]. The difference in susceptibility to Cry1A toxins in the Europe-R ECB strain (Cry1Ab resistant) has been linked to an altered receptor binding which was suggested by the reduced concentration of cadherin receptors in the resistant strain as compared to susceptible strains [19]. In the same study, however, the other Cry1Ab resistant ECB strain (RSTT-R) did not exhibit a reduction in cadherin expression and the authors suggested that other factors may have more important contributions to resistance in this strain [19].

In a recent expressed sequence tag (EST) analysis in the gut of the ECB larvae [26], we identified 10 cDNAs putatively encoding aminopeptidase-like proteins which are reported to be candidate receptors of Cry toxins. The main objective of the present study was to explore the possible involvement of these genes in Bt toxicity and resistance in the ECB. Our results suggest that the cDNA which encodes aminopeptidase P-like protein is involved in Cry1Ab toxicity and in resistance to Cry1Ab in the ECB.

## Results

### Sequencing and analysis of cDNAs encoding aminopeptidase-like proteins

**Figure 1:**
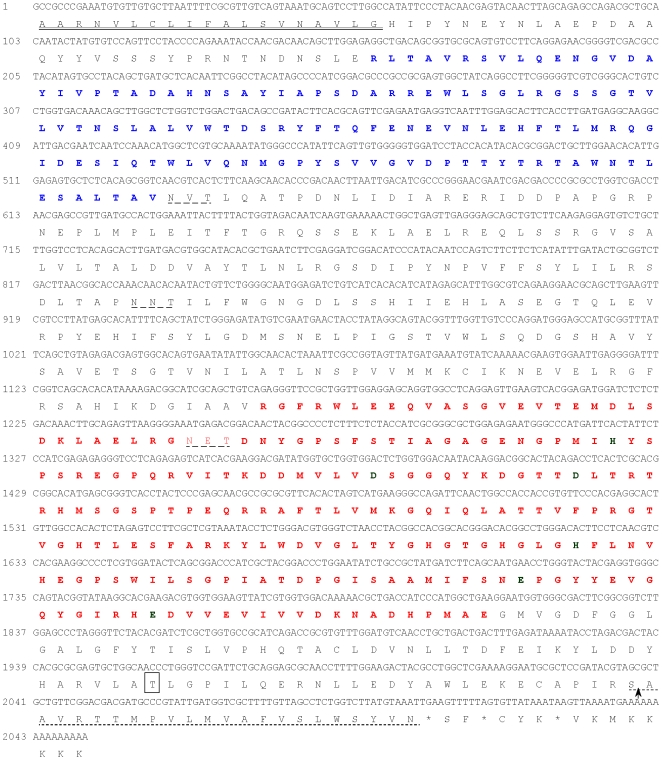
Sequence analysis of aminopeptidase P-like (*OnAPP*) gene from the Europeans corn borer larvae. The putative N-terminal signal peptide is double underlined. Two domains for OnAPP were predicted using smart software [55]. The Creatinase/Prolidase N-terminal domain is bold and blue color (residues 53 – 177), and metallopeptidase family M24 domain is bold and red color (residues 372 – 603). Six active site residues within metallopeptidase domain are bold and dark red color. The GPI-anchored signal peptide is dot-underlined and the possible cleavage site of anchor moiety is indicated by arrow. The predicted O-glycosylated residue is boxed and the putative N-glycosylation sites are dash-underlined.

We searched our gut-specific ECB EST database, which consisted of 15,000 ESTs [26], and revealed 10 ESTs that shared similarities to known aminopeptidases (E-value ≤10^−3^). Nine of the ESTs (*OnAPN1* to *OnAPN9*) showed similarities to aminopeptidase-N (APN) like genes, and one EST (*OnAPP*) showed similarity to an aminopeptidase-P (APP) like gene (Table 1). APP is a metalloprotease that releases the N-terminal amino acid residue from peptides with a penultimate proline residue. Previous analysis of our EST database identified 13 ESTs with similarities to aminopeptidase-like genes but further analysis from the 3′prime end sequencing of the clones reduced the number to 10 [26]. Among the APN ESTs, four sequences showed 94-98% identities with ECB sequences already deposited in the NCBI database by Coates et al. [27] (Table 1). These clones have insert sizes ranging from 679 – 2143 bp. The ESTs putatively encoding APN have percent identities of 38 - 98% with other known APN. The single *OnAPP* cDNA showed the highest identity (42%) with the APP from *Tribolium casteneum.* Further analysis of the *OnAPP* gene predicted that the signal peptide cleavage site occurred after Gly-19 according to Hidden Markov models. This gene was likely to encode a membrane bound protein as glycosylphosphatidyl-inositol (GPI)-anchor signal was predicted at the C terminal end of this sequence (Figure 1). OnAPP also had three potential *N*-glycosylation sites at residues N178, N278 and N417 and one *O*-glycosylation site at residue T653. The predicted molecular mass of the mature OnAPP protein is 72.7 kDa and it has a pI of 4.82. The *OnAPP* cDNA and its deduced amino acid sequences have been deposited in the NCBI database with the accession number of JF262040.

**Table 1:** Aminopeptidase-like genes identified from *Ostrinia nubilalis* larval gut EST database.

Gene name	Length (bp)	Matches	Organism	Aminopeptidase type	Identity	E-value	Score (bits)
*OnAPP*	2155	XP_974698.1	*Tribolium castaneum*	Aminopeptidase P	42%	2e-124	451
*OnAPN1*	924	ABL01482.1	*Ostrinia nubilalis*	Aminopeptidase N2	95%	2e-30	139
*OnAPN2*	991	ABL01481.1	*Ostrinia nubilalis*	Aminopeptidase N1	98%	6e-105	386
*OnAPN3*	2057	ABL01483.1	*Ostrinia nubilalis*	Aminopeptidase N3	94%	1e-161	574
*OnAPN4*	880	AAK85538.1	*Helicoverpa armigera*	Aminopeptidase N	38%	2e-27	128
*OnAPN5*	836	ABQ51393	*Ostrinia furnacalis*	Aminopeptidase N	70%	4e-73	280
*OnAPN6*	1904	ACA35025	*Helicoverpa armigera*	Aminopeptidase N6	65%	3e-124	450
*OnAPN7*	829	ABL01484.1	*Ostrinia nubilalis*	Aminopeptidase N4	98%	1e-88	332
*OnAPN8*	1180	AAP37951.1	*Helicoverpa armigera*	Aminopeptidase N2	68%	4e-99	351
*OnAPN9*	679	ABL01483	*Ostrinia furnacalis*	Aminopeptidase N3	75%	3e-67	261

### Tissue and developmental stage specific expression of aminopeptidase-like genes

**Figure 2:**
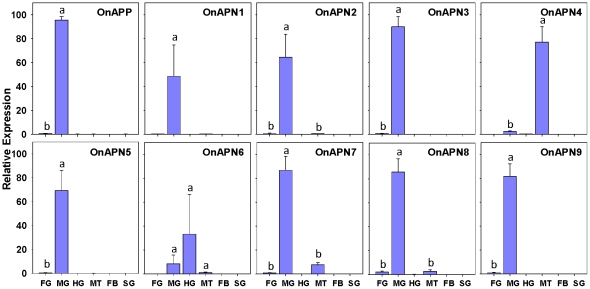
Expression profiles of 10 aminopeptidase-like genes in larval tissues of 1-day-old Cry1Ab-susceptible European corn borer. Gene expression was determined in foregut (FG), midgut (MG), hindgut (HG), Malphigian tubules (MT), fatbodies (FB), and salivary glands (SG) by real-time PCR. The ribosomal protein S3 (*RPS3*) gene was used as a reference gene to calculate the relative expression levels. Standard error bars were determined from three biological replications and two technical replications. Different letters within a figure represent significant difference at *P* value≤0.05.

The mRNA level was assessed for all 10 aminopeptidase-like genes in six different tissues of Bt susceptible 1-day old fifth-instar larvae using real-time quantitative PCR (qPCR) (Figure 2). There was no detectable expression in fatbodies and salivary glands for any of the 10 genes. The expression of these genes was predominantly in midgut tissues except for *OnAPN4* which had highest expression in Malphigian tubules and *OnAPN6* exhibited highest expression in hindgut. Very low transcript levels were observed in the foregut for most of the genes except *OnAPN1*, *OnAPN4* and *OnAPN6* that had no detectable expression. In addition to *OnAPN4*, three other genes, *OnAPN6*, *OnAPN7*, and *OnAPN8*, have detectable expression in Malphigian tubules.

**Figure 3:**
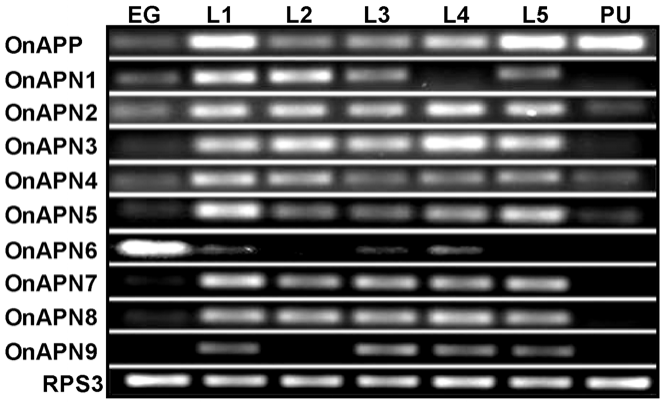
Expression profiles of 10 aminopeptidase-like genes in seven developmental stages of Cry1Ab-susceptible European corn borer. Egg (EG), first instar (L1), second instar (L2), third instar (L3), fourth instar (L4), fifth instar (L5) larvae, and pupae (PU). The ribosomal S3 protein (*RPS3*) gene was used as a reference gene.

The expression of all 10 aminopeptidase-like genes from the ECB was assessed by RT-PCR in different developmental stages including eggs, five larval instars, and pupae (Figure 3). Most of the genes had high transcript expression in the larval stages except for *OnAPN6* which was expressed predominantly in eggs but limited expression in the first-, third-, and fourth-instar larvae. In addition, transcripts of the *OnAPP*, *OnAPN2*, *OnAPN4*, and *OnAPN5* were detected in pupae, even though band intensity for *OnAPN2*, *OnAPN4*, and *OnAPN5* was lower than for the larval stage. *OnAPP* has expression in all the developmental stages with the highest expression in the first- and fifth-instar larvae and pupae. The expression of this gene was high in the egg and first instar, then was low in the second instar and remained low until pupation. *OnAPN1* had the highest expression in the first and second instars and its transcript was detected in eggs, third- and fourth-instar larvae. Transcripts of *OnAPN2*, *OnAPN4* and *OnAPN5* were detected in all developmental stages, but transcripts of *OnAPN3*, *OnAPN7*, *OnAPN8* and *OnAPN9* were only detected in the larval stage.

### Analysis of aminopeptidase-like genes in resistant and susceptible strains of ECB

**Figure 4:**
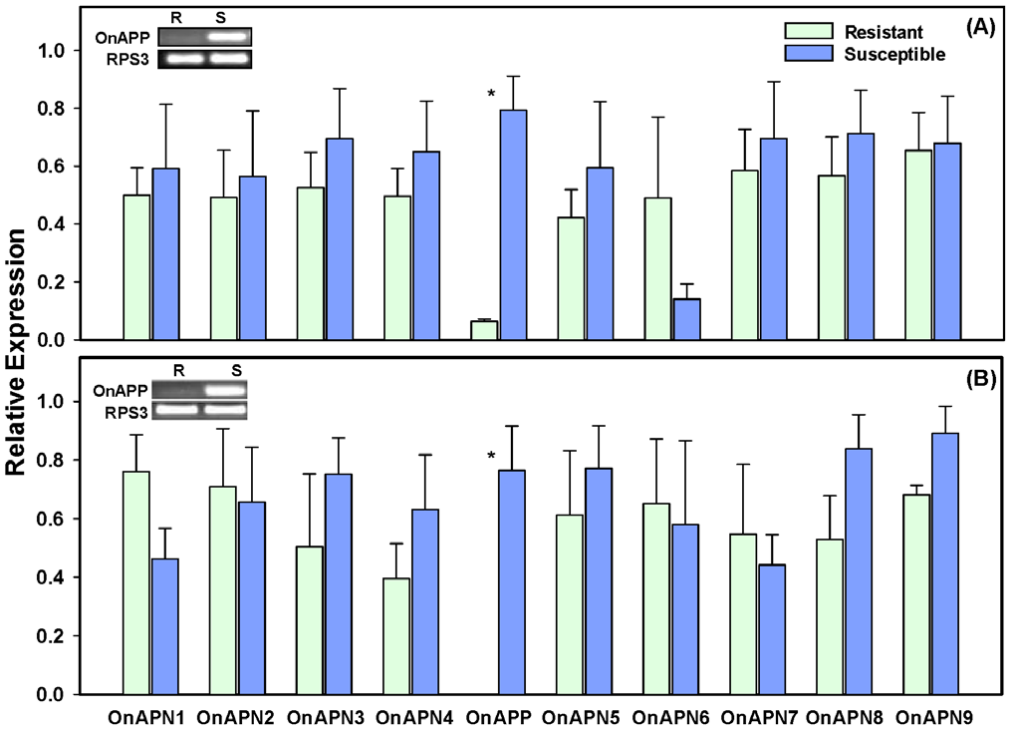
Expression profiles of 10 aminopeptidase-like genes in the larvae of Cry1Ab resistant and susceptible strains of European corn borer. Expression data was generated from two pairs of resistance and susceptible strains: (A) RSTT-R and Europe-S and (B) SKY-R and KY-S. Bars represent relative expression for a particular gene for resistant and susceptible ECB strains based on real-time PCR values. There were three biological replications and two technical replications. Asterik (*) indicates the significant difference at *p* value <0.01. Gel picture for RT-PCR for the *OnAPP* gene is given in the upper left corner. *RPS3* gene was used as a reference gene.

**Figure 5:**
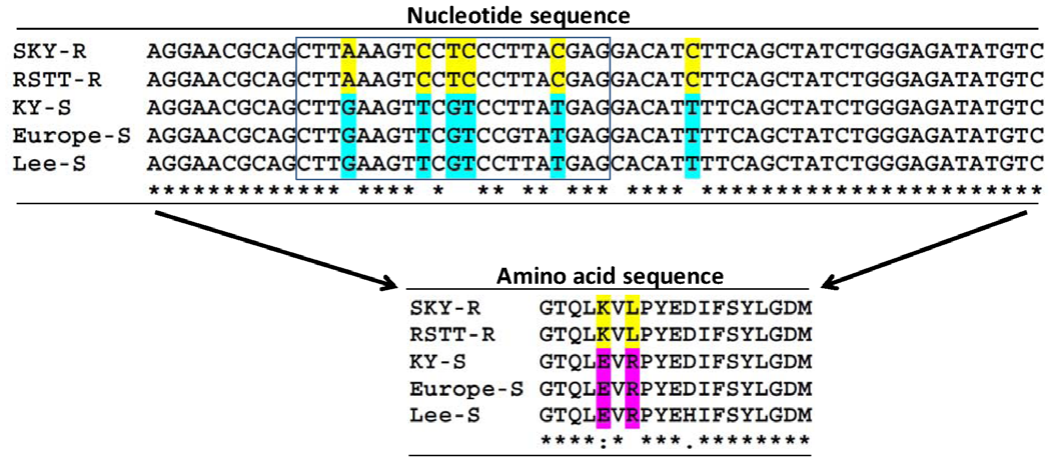
Comparisons of nucleotide and amino acid sequences between two resistant strains (SKY-R and RSTT-R) and three susceptible strains (KY-S, Europe-S, and Lee-S) of European corn borer. The rectangular block on the nucleotide sequence indicates the forward primer.

**Figure 6:**
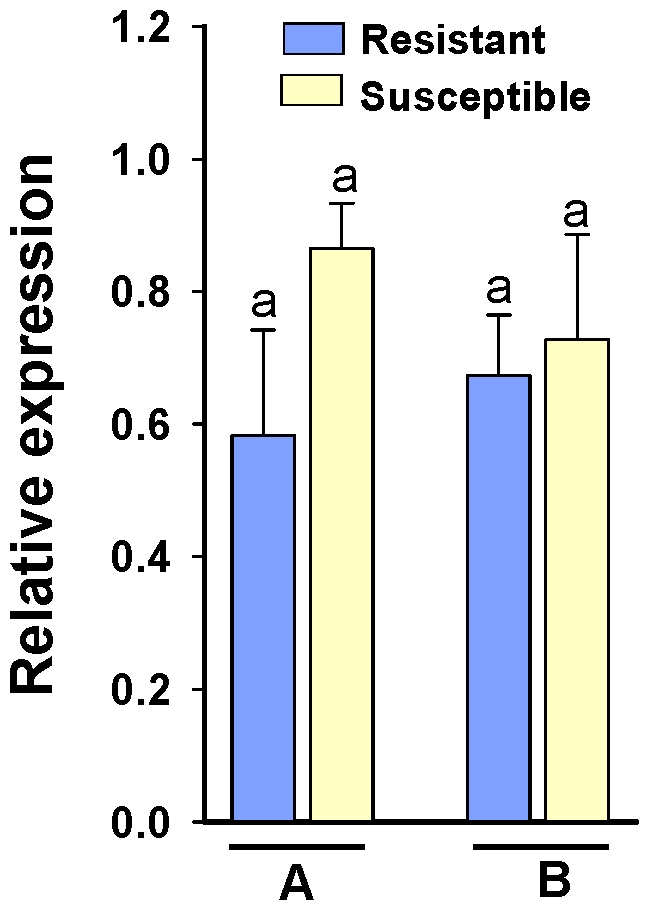
Expression profiles of *OnAPP* gene in the larvae of Cry1Ab resistant and susceptible strains of European corn borer by using second set of primers (OnAPP_P2). Expression data was generated from two pairs of resistant and susceptible strains: (A) SKY-R and KY-S, and (B) RSTT-R and Europe-S. Bars represent relative expression for a particular gene for resistant and susceptible ECB strains based on real-time PCR values. The data were based on three biological replications; each with two technical replications. *RPS3* gene was used as a reference gene.

To identify the aminopeptidase-like genes which may have a potential role in the Bt toxicity and resistance, we analyzed the expression of these genes in two pairs of Cry1Ab resistant and susceptible ECB strains (Figure 4). Our analysis showed that except for *OnAPP*, the other genes exhibited similar levels of the transcript in Cry1Ab resistant and susceptible strains of ECB. In order to make sure that the expression difference of *OnAPP* was not due to mutations in the gene, we sequenced 678 bp region of the gene containing the primer sequences from both strains. We found several nucleotides in the forward primer sequence that differed between the resistant and susceptible larvae (Figure 5). This difference was consistent across the two resistant strains and the three susceptible strains. The translated amino acid sequence of this region had two amino acid residues that differed between resistant and susceptible ECB larvae (Figure 5). At position 305, the glutamic acid residue (E) was changed to lysine (K) and at position 307, the arginine residue (R) was changed to leucine (L) in resistant larvae as compared with the susceptible larvae. We also assessed the expression of *OnAPP* using different set of primers and found no difference in its expression between resistant and susceptible larvae (Figure 6).

### RNA interference of aminopeptidase-P like gene

**Figure 7:**
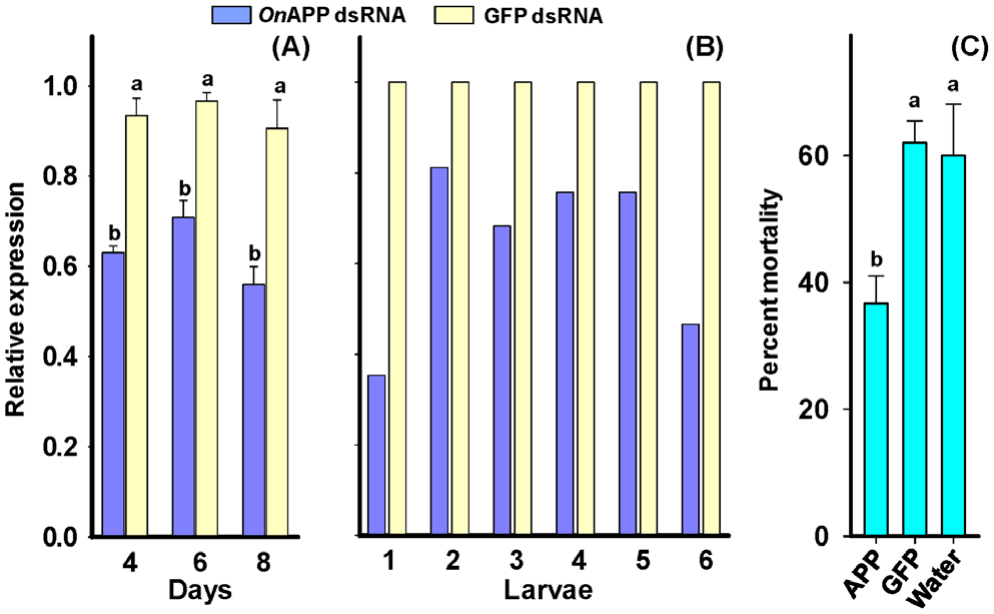
The expression of *OnAPP* gene in the larvae fed *OnAPP* dsRNA and GFP dsRNA. Immediately after the larvae developed into second instar, they were individually fed fresh artificial ECB diet containing dsRNA. (A) The expression of *OnAPP* gene in *OnAPP* dsRNA and GFP dsRNA treated larvae after 4, 6, and 8 days. Standard errors of the means were determined from three biological replications, each with two technical replications. (B) The expression of *OnAPP* for six individual midguts after 8 days of dsRNA feeding. (C) Percent mortality in *OnAPP* dsRNA, GFP dsRNA , and water treated larvae. Standard errors of the means were determined from three replications; each replication consisted of 50 larvae. Different letters represent significant difference with *p* value ≤0.05.

To confirm the potential role of the *OnAPP* gene in Bt toxicity in ECB larvae, we developed a feeding-based RNA interference (RNAi) technique to silence the expression of the *OnAPP* gene. Immediately after the larvae developed into the second instar, they were fed an artificial diet mixed with *OnAPP* dsRNA. The dsRNA for green fluorescent protein (GFP) gene was used as control. After 4, 6 and 8 days, larvae were dissected to obtain midguts. Four midguts were pooled as a sample to assess the mRNA level in larvae fed diets containing *OnAPP* dsRNA or *GFP* dsRNA. The transcript level for the *OnAPP* gene was reduced by 32.5, 26.6, and 38.2% after 4, 6, and 8 days, respectively, as compared with the larvae fed *GFP* dsRNA. This indicated that there was a statistically significant reduction in *OnAPP* mRNA levels in *OnAPP* dsRNA-fed larvae (Figure 7A). In order to determine how the dsRNA feeding affects the *OnAPP* mRNA in individual larvae, we performed the same experiment except that *OnAPP* transcript levels were determined in individual larvae. We found that expression of the *OnAPP* gene was reduced from 18.8 – 64.7 % in *OnAPP* dsRNA treated larvae as compared with *GFP* dsRNA treated larvae (Figure 7B). We also exposed larvae that were fed artificial diet containing *OnAPP* dsRNA, *GFP* dsRNA, and water to the artificial diet containing Cry1Ab toxin for 7 days (Figure 7C). We found that mortality of larvae decreased by 23 and 25% in the *OnAPP* dsRNA treatment as compared with that in the *GFP* dsRNA or water treatments (Figure 7C).

## Discussion

The genetic basis of insect resistance to Bt toxins has been suggested to be multigenic. Insects can develop resistance due to reduced solubilization, deficient proteolytic activation, increased proteolytic degradation, sequestration of toxin molecules by non-functional binding sites, changes in functional binding sites, defective pore formation and enhanced cellular repair [28]–[31]. In several studies, altered binding sites have been found to associate with high levels of Bt resistance in insects. In the ECB, there may be more than one independent resistance mechanism [32]. Indeed, the resistance in the two ECB strains (RSTT-R and SKY-R) has been reported to be polygenic in nature [33], [34]. In Dipel-resistant ECB strain, the resistance mechanism has been associated with the reduced protease level in resistant larvae as compared with the susceptible larvae [23]. However, Cry1Ab resistant and susceptible strains of ECB had no consistent differences in activities of midgut proteases [25], although a reduction in cadherin receptors in the resistance strain (Europe-R) as compared with the susceptible strain was observed [19]. Similar results were not found for the other Cry1Ab resistant strain (RSTT-R) in the same study.

These results suggest that resistance in ECB could be due to changes in the midgut receptors which affect its binding with the Cry toxin [19]. A recent study in ECB found no association between ECB Cry1Ab resistance with segregation of APN1, bre5 (Onb3GalT5), and cadherin alleles in a Cry1Ab resistant ECB colony [32]. These reports suggest that there may be other proteins that play important roles in Bt resistance in ECB. In other insects, several Cry toxin receptors have been reported such as cadherin, GPI anchored aminopeptidase N, GPI anchored alkaline phosphatase, GPI-ADAM metalloprotease, glycolipids, glyco-conjugate, V-ATP synthase subunit, and actin [5].

In this study, we identified and analyzed 10 aminopeptidase-like genes in Cry1Ab resistant and susceptible strains and found that aminopeptidase P-like gene was most likely to be associated with Bt toxicity and resistance in ECB. Gene expression analysis for 10 aminopeptidase-like genes revealed that most of these genes were expressed predominantly in midgut tissues except for *OnAPN4* and *OnAPN6* which were expressed predominantly in the Malphigian tubules and hindgut, respectively. No expression was detected for any gene in fatbodies and salivary glands. These results are consistent with expression analysis of the aminopeptidase N genes in *O. nubilalis* (35), *Trichopluisa ni* [36] and *Helicoverpa armigera* [37] where all genes were expressed predominantly in midgut tissues. In *T. ni,* however, two of the APN genes (APN1 and APN2) were also detected in Malphigian tubules and no expression was detected in fatbodies or salivary glands [36]. In *Achaea janata* and *Spodoptera litura,* novel GPI anchored aminopeptidase N like genes were detected in the fatbodies with no expression in midgut tissues [38], [39]. We did not find any expression of aminopeptidase in the fatbodies; this may be because our cDNA library was constructed from the gut of the ECB. The aminopeptidase N genes in the midgut would have roles in the peptide digestion with various N-terminal residues [40]–[42], but its role in Malphigian tubules and fatbodies is unclear.

It has been suggested that fatbody APN may play a significant role in metamorphosis [38] whereas APN expression in Malphigian tubules may have a role in the hydrolysis of peptides in the tubule lumen [36]. The expression of ECB aminopeptidase-like genes was associated primarily with larval stages except for *OnAPN6* which had the highest expression in eggs, suggesting that it may have a role in embryonic development. The *OnAPP* gene was highly expressed in pupae as well as in the first and fifth instars. This expression pattern is similar to a cytosolic APP from *Drosophila melanogaster,* where APP protein can be detected in the larval stage and its signal increases in pupae [43].

Our gene expression analysis of Cry1Ab-resistant and -susceptible strains revealed that there were no differences in the expression of aminopeptidase-like genes between resistant and susceptible strains. Similar results for two resistant strains (RSTT-R and SKY-R) and two susceptible (Europe-S and KY-S) strains suggest that altered expression of these genes is unlikely to be responsible for resistance. However, these results do not rule out the possibility that mutations in the sequences of these genes in resistant larvae may play a role in resistance. Indeed, we found several nucleotide changes in the region from 912 to 930 bp of the *OnAPP* gene and these changes in nucleotide sequence were similar in the two resistant strains causing changes in two amino acids, Glu^305^ to Lys^305^ and Arg^307^ to Leu^307^. Mutations in the receptor genes like cadherin and APN have been reported to be associated with Cry toxin resistance in several insects. In *H. virescens* Cry1Ac-resistant line YHD2, a single mutation in cadherin gene has been shown to be responsible for 40–80% of Cry1Ac resistance levels [14]. Similarly, different mutated cadherin alleles were associated with Cry1Ac resistance in *P. gossypiella* and *H. armigera* [15], [44]. Mutation of the APN gene in *H. armigera* has also been reported to be associated with Cry1Ac resistance [17]. In addition, a recent study showed that a mutation in the ABC transporter gene (ABCC2) is correlated with insect resistance to Cry1Ac toxin [45]. These authors suggested that in addition to the two binding steps (binding to cadherin protein and binding to GPI-anchored protein) as proposed in the pore formation model, there is an additional binding step. In this step Cry1Ab and Cry1Ac, as pre-formed oligomers or possibly also as monomers bind to the open configuration of ABCC2 which facilitates subsequent membrane insertion [45]. This and other recent studies have shown that the mode of action of Bt toxins [45] and thus resistance mechanisms are more complex than they have been previously envisioned.

To date, APP-like genes had not been implicated in the Bt toxicity and resistance. To our knowledge this is the first report where APP-like gene from an insect with predicted GPI-anchor signal peptide at the C-terminal has been identified. We found only one report from *D. melonogaster* where a cytosylic form of APP had been characterized [43]. We also searched the NCBI database to find any APP with potential GPI anchor signal from insects but no results were found. APP is a metalloprotease that releases the N-terminal amino acid residue from peptides with a penultimate proline residue [46]. While the physiological role of APP in insects is unclear, mammalian APP is involved in the protein turnover of collagen and the regulation of biologically active peptides, such as substance P and bradykinin [47]–[49]. Cry proteins have the ability to bind with receptors that are anchored to the membrane via a GPI moiety, which facilitates membrane insertion and pore formation [5], but weather GPI anchored APP in ECB is a receptor of the Cry1Ab toxin will deserve further investigation.

Further evidence for the role of OnAPP in Cry1Ab toxicity is provided by gene silencing experiments. The expression of the *OnAPP* gene in susceptible ECB larvae was reduced by 38% after larvae fed *OnAPP* dsRNA for 8 days. But our expression data using individual midguts revealed that there was a lot of variation among individuals in the reduction of *OnAPP* transcript following the dsRNA feeding. This variation may be due to the difference in the ability of individuals to ingest different amount of dsRNA or may be due to the ability of individual insects to degrade the dsRNA in the midgut. Our Bt bioassay using insects fed *OnAPP* and *GFP* dsRNA resulted in reduced susceptibility of the larvae to Cry1Ab by 23–25 %. These data suggest that *OnAPP* gene may play an important role in Cry1Ab toxicity in the ECB. Further experiments will be needed to determine the precise nature of this mechanism. The low reduction in the percent susceptibility in *OnAPP* dsRNA treated insects may also be due to the small reduction of *OnAPP* transcript following dsRNA treatment and the high variation of *OnAPP* transcript level among individuals. This also suggests that the *OnAPP* gene may not be solely responsible for resistance in the ECB and there may still be other factors that may contribute to Bt resistance. Nevertheless, our results strongly suggest that *OnAPP* gene is a good candidate for further study to elucidate the Bt toxicity and to understand the mechanism of resistance in ECB.

## Materials and Methods

### Insects rearing

The Bt-susceptible European corn borers (Lee-S) used in this study for tissue and developmental stage expression and also for RNAi experiments were purchased as eggs and larvae (Lee French Laboratories, Lumberton, MN). The Nebraska resistant ECB (SKY-R) strain originated from a field collection of 126 diapausing larvae obtained from non-Bt hybrids in Kandiyohi Co., MN in 2001. The resistant strain was initiated from 14 larvae that survived exposure to a diagnostic Cry1Ab concentration used to identify potential changes in susceptibility to Cry1Ab [33], [50]. To minimize inbreeding or founder effects, the resistant insects were backcrossed twice with the susceptible strain which originated from the same collection. Because the resistance was incompletely recessive and involved multiple factors [33], the F1 progeny were randomly mated to obtain recombination of resistance factors in the F2 progeny to allow selection of resistant genotypes. The insects were then subjected to selection at a Cry1Ab concentration corresponding to 2- to 3-fold the LC_50_ for the F1 progeny (150 ng/cm^2^) [51]. This selection event was designed to eliminate all the susceptible homozygotes and most of the heterozygotes.

The resistant survivors from this selection event were then subjected to a second cycle of backcrossing, random mating, and selection. After six generations, the Cry1Ab concentration used in selections was gradually increased to achieve 750 ng/cm^2^ at generation F10, a concentration that kills virtually all F1 progeny. At generation F17, the resistance to Cry1Ab in the re-selected strain was in excess of 800-fold. Populations of ECB designated Europe-S and RSTT-R were established as laboratory strains. The European strain was established in 1993 from approximately 500 ECB larvae collected in the Lombardia region of northern Italy and was provided to the University of Nebraska after 20 generations of laboratory rearing. This strain was divided into two subpopulations, one exposed throughout development to Cry1Ab protoxin from the *B. thuringiensis* subsp. *kurstaki* strain HD1–9, which produces only Cry1Ab protein (Europe-R), and the other reared in the absence of protoxin (Europe-S). The RSTT-R strain resulted from a combination of individuals from the Europe-R selection line and from a selection line from insects collected in Nebraska [20].

The guts were dissected from fifth-instar larvae in DEPC (diethylpyrocarbonate)-treated distilled water and were stored in TRI reagent™ (Molecular Research, Inc., Cincinnati, OH) at −80°C until used.

### cDNA sequence analysis

A gut-specific EST library was established from RNA isolated from fifth-instar ECB larvae as previously described and 15,000 clones were sequenced [26). The EST database consisting of 2,895 unique ESTs was searched for the genes encoding aminopeptidase-like genes. Ten clones from our EST library were identified, nine similar to aminopeptidase N and one similar to aminopeptidase P like genes. These clones were again sequenced from both ends using M13R and M13F primers in order to determine that these genes were unique. Signal P software was used to predict signal peptide [52]. The software ClustalW [53] was used for multiple alignments and PredGPI was used to predict GPI anchor signal [54]. N-glycosylation sites were predicted by NetNGlyc 1.0 (http://www.cbs.dtu.dk/services/NetNGlyc/) and O-glycosylation sites were predicted by NetOGlyc 3.1 (http://www.cbs.dtu.dk/services/NetOGlyc/) [55]. Smart software [56] was used to predict domains in the amino acid sequences.

### Tissue and developmental stage expression profiles

The feeding larvae of Cry1Ab susceptible colony (from Lee French Laboratories, Lumberton, MN) were used in this analysis. Tissues were dissected in DEPC-treated water from one-day-old fifth-instar ECB larvae. Total RNA was isolated from different tissues (pooled from four animals) and different ECB developmental stages (pooled from four animals) using TRI reagent™ (Sigma, St. Louis, MO) and treated with TURBO™ DNase (Ambion, Austin, TX) to remove any genomic DNA contamination. One microgram of total RNA was used for synthesis of first strand cDNA using SuperScript® III First-Strand Synthesis System (Invitrogen, Carlsbad, CA). cDNA prepared from total RNA was used as a template for real-time qPCR or RT-PCR. PCR primers were designed using Beacon Designer software (version 7) and sequences were blast search to eliminate the occurrence of cross reactions. The qPCR analysis was performed using SYBR green kit (Bio-Rad) and Bio-Rad iCycler iQs real-time PCR detection system at the Kansas State University Gene Expression Facility. qPCR cycling parameters included 95°C for 5 min, 40 cycles each consisting of 95°C for 30 sec, 55°C for 0.15 sec, and 72°C for 0.45 sec, followed by 95°C for 1 min and 55°C for 1 min. At the end of each quantitative PCR experiment, a melt curve was generated to confirm single peak and rule out the possibility of primer-dimer and non-specific product formation. Efficiencies for each pair of primers were calculated using four 5-fold serial dilutions (1∶1, 1∶5, 1∶25, and 1∶125) in triplicates. Amplification efficiencies were higher than 97% for all primer pairs used in this study. All primer combinations used in this study showed a linear correlation between the amount of cDNA template and the amount of PCR product. All correlation coefficients was larger than 0.99 (Table 2, Figure S1). iCycler software (Bio-Rad Laboratories) was used to determine slope, efficiency, and correlation co-efficient for each primer pairs and are provided in the Table 2. Relative fold-changes for transcripts were calculated using the comparative 2^−ΔΔCT^ method (57) and normalized to RPS3 gene [35], [58]-[60] and statistical analysis was performed using the relative expression software tool (REST V2.0.13) (Qiagen , Valencia, California, USA) [61]. For RT-PCR, 27 cycles were used for all genes including RPS3 gene, each cycle consisting of 94°C for 30 s, 55°C for 60 s, and 72°C for 60 s. For qPCR analysis, there were three biological replications, each with two technical replications.

**Table 2:** Sequences and relevant parameters for each primer pairs used for expression profiling of 10 aminopeptidase-like genes in *Ostrinia nubilalis* by qPCR.

Gene name	Forward primer	Reverse primer	PS (bp)	CC	Slope	PE (%)
*OnAPP_P1*	CTTGAAGTTCGTCCTTATGAG	CACTGTGCCACTCGTCTC	138	1.000	−3.327	99.8
*OnAPN1*	ACCCTAACAGTAAGACAGTTTGAC	TGGCACTACAAGCAAGTAACG	197	0.997	−3.387	97.4
*OnAPN2*	TCTGTAGTCTGGTTCACATTATCC	ACTCACCTCCGCTGTATCC	84	0.998	−3.350	98.8
*OnAPN3*	CTTCAACAGCCCACTGGAGAG	ACGCAAGACATATTAGGTAACAGC	85	0.999	−3.356	98.6
*OnAPN4*	ATCTGAAAAGCACCAACAGTCTTC	CTCTCGCCCTGATCGTCTTATG	156	0.999	−3.356	98.6
*OnAPN5*	TGTATTGGCGGAGTCTGATTC	CCAGTCGTCATTGAGGAACC	93	0.998	−3.308	100.6
*OnAPN6*	GCACCCCATTCATTGTTCGC	GTATCTGGACGAGCCTGGAC	126	0.996	−3.014	114.7
*OnAPN7*	AATTCCAAACCTGGGCGTAC	GTTGTTCATGGCACTGTTGAC	89	0.998	−3.342	99.2
*OnAPN8*	AAGTCGTAAAGAGTAAACTGAGAG	GCCAGATCCAGCATGAAGTG	112	0.998	−3.324	99.9
*OnAPN9*	CGCCGTGACCGTAACTGG	GTCGTCGCTAACAGAGAAGAG	93	0.999	−3.307	100.6
*OnAPP_P2*	CCA GCG AGA TCG TGT AGA AC	GTG GAA GTT ATC GTG GTG GAC	106	0.999	−3.359	98.5

PS: Product size; CC: Correlation coefficient; PE: Primer efficiency.

### Expression profiles in Cry1Ab resistant and susceptible larvae

Transcript level for all 10 aminopeptidase-like genes were assessed in the midgut tissues from fifth-instar larvae from each strain (Cry1Ab-susceptible and -resistant strains). Total RNA was isolated from four midguts pooled together using TRI reagent™ (Sigma, St. Louis, MO), and treated with TURBO™ DNase (Ambion, Austin, TX) to remove any genomic DNA contaminations. First strand cDNA preparation, and qPCR analysis were performed as described above. For qPCR analysis there were three biological replications, each with two technical replications.

### RNA interference

The design of the *OnAPP* RNAi construction is illustrated in Figure S2. The dsRNA was prepared using the plasmid DNA as template by *in vitro* transcription for RNAi. The primers were designed using Beacon Designer software (version 7). T7 primer sequence was placed in front of both forward and reverse primers. The primer sequences to generate dsRNA for *OnAPP* gene were 5′- TAATACGACTCACTATAGGGTTTGGTCCT CACAGCACTTG and 3′- TAATACGACTCACTATAGGGTTCACTGTGCCACTCG TCTC with product size of 333 bp. Similarly, for GFP, the primers used were 5′- TAATACGACTCACTATAGGCCATTCTTTTGTTTGTCTGC and 3′- TAATACGACTCACT ATAGGGGCCAACACTTGTCAC with product size of 309 bp. The dsRNA was transcribed using the above gene specific primers and the AmpliScribe™ T7-Flash™ Kit (Epicentre Technologies, Madison, WI) according to the manufacturer's protocol. The dsRNAs were purified by phenol/chloroform extraction followed by ammonium acetate precipitation. Immediately after the development of larvae into second instar, they were individually fed the dsRNA mixed with fresh artificial ECB diet (Bio-serve). Three doses, each consisting of 10 µg of *OnAPP* dsRNA in 2 µl of water were added to the diet for each larva on day 0, 2, and 4 for a total of 30 µg dsRNA per larva. The control larvae received the same amount of GFP dsRNA or equal volume of water. After day 6 (2 days after last dsRNA treatment), larvae were transferred to normal artificial diet. Transcript levels of *OnAPP* in the midgut tissues of the larvae fed *OnAPP* and GFP dsRNA were determined by qPCR on day 4, 6 and 8 after first dsRNA treatment. Total RNA isolation, first strand cDNA preparation, and qPCR analysis were performed as described above. Three biological replications, each with two technical replications, were used for qPCR analysis.

To perform Cry1Ab bioassay, the RNAi experiment was performed as above and on day 6, larvae were exposed to Cry1Ab toxin at 2 µg/ml of diet and allowed to feed for 7 days. The mortality of the larvae was recorded on 7^th^ day after Bt treatment. Fifty larvae were used in each experiment and three independent experiments were performed for this bioassay.

### Statistical analysis

The gene expression and mortality analysis were subjected to one-way analysis of variance (ANOVA). Fisher's least significant difference (LSD) multiple comparisons were then used to separate the means among the treatments. All the statistical analyses were performed using ProStat software (Poly Software International Inc., Pearl River, NY).

## Supporting Information

Figure S1 **Primer efficiency test by qPCR.** Efficiencies of each pair of primers were calculated using four 5-fold serial dilutions (1∶1, 1∶5, 1∶25, and 1∶125) in triplicates. iCycler software (Bio-Rad Laboratories) was used to determine slope, efficiency, and correlation co-efficient for each primer pairs. Summarized data are provided in Table 2. Correlation coefficients for all the primers are >0.99.
(PDF)

Figure S2 **The design of the OnAPP dsRNA construction.** The fragment (F) indicates the region used to generate the double stranded RNA for RNAi experiment. Only Cry1Ab-susceptible ECB larvae were used in this analysis. P1 and P2 indicated the location of primer set 1 and primer set 2 used to assess the expression of *OnAPP* in this study.
(TIF)

## References

[1] Schnepf E, Crickmore N, Van Rie J, Lereclus D, Baum J (1998). *Bacillus thuringiensis* and its pesticidal crystal proteins.. Microbiol Mol Biol Rev.

[2] Federici BA (2005). Insecticidal bacteria: an overwhelming success for invertebrate pathology.. J Invertebr Pathol.

[3] Valaitis, AP, Jenkins JL, Lee MK, Dean DH, Garner KJ (2001). Isolation and partial characterization of gypsy moth BTR-270, an anionic brush border membrane glycoconjugate that binds *Bacillus thuringiensis* Cry1A toxins with high affinity.. Arch Insect Biochem Physiol.

[4] Shelton AM, Zhao JZ, Roush RT (2002). Economic, ecological, food safety, and social consequences of the deployment of Bt transgenic plants.. Annu Rev Entomol.

[5] Soberon M, Gill SS, Bravo A (2009). Signaling versus punching hole: how do *Bacillus thuringiensis* toxins kill insect midgut cells?. Cell Mol Life Sci.

[6] Bravo A, Gomez I, Conde J, Munoz-Garay C, Sanchez J (2004). Oligomerization triggers binding of a *Bacillus thuringiensis* Cry1Ab pore-forming toxin to aminopeptidase N receptor leading to insertion into membrane microdomains.. Biochim Biophys Acta.

[7] Gill SS, Cowles EA, Pietrantonio PV (1992). The mode of action of *Bacillus thuringiensis* endotoxins.. Annu Rev Entomol.

[8] Zhang X, Candas M, Griko NB, Rose-Young L, Bulla LA (2005). Cytotoxicity of *Bacillus thuringiensis* Cry1Ab toxin depends on specific binding of the toxin to the cadherin receptor BT-R1 expressed in insect cells.. Cell Death Differ.

[9] Krishnamoorthy M, Jurat-Fuentes JL, McNall RJ, Andacht T, Adang MJ (2007). Identification of novel Cry1Ac binding proteins in midgut membranes from *Heliothis virescens* using proteomic analyses.. Insect Biochem Mol Biol.

[10] Ochoa-Campuzano C, Dolores Real MD, Martínez-Ramírez AC, Bravo A, Rausell C (2007). An ADAM metalloprotease is a Cry3Aa *Bacillus thuringiensis* toxin receptor.. Biochem Biophys Res Commun.

[11] Pigott C, Ellar DJ (2007). Role of receptors in *Bacillus thuringiensis* crystal toxin activity.. Microbiol Mol Biol Rev.

[12] Ferre J, Van Rie J (2002). Biochemistry and genetics of insect resistance to *Bacillus thuringiensis.*. Annu Rev Entomol.

[13] Oppert B, Kramer KJ, Beeman RW, Johnson D, McGaughey WH (1997). Proteinase-mediated insect resistance to *Bacillus thuringiensis* toxins.. J Biol Chem.

[14] Gahan LJ, Gould F, Heckel DG (2001). Identification of a gene associated with *Bacillus thuringiensis* resistance in *Heliothis virescens.*. Science.

[15] Morin S, Biggs RW, Sisteson MS, Shriver L, Ellers-Kirk C (2003). Three cadherin alleles associated with resistance to *Bacillus thuringiensis* in pink bollworm.. Proc Nat Acad Sci.

[16] Soberon M, Pardo-Lopez L, Lopez I, Gomez I, Tabashnik B (2007). Engineering modified Bt toxins to counter insect resistance.. Science.

[17] Zhang S, Cheng H, Gao Y, Wang G, Liang G (2009). Mutation of an aminopeptidase N gene is associated with *Helicoverpa armigera* resistance to *Bacillus thuringiensis* Cry1Ac toxin.. Insect Biochem Mol Biol.

[18] Rajagopal R, Sivakumar S, Agrawal N, Malhotra P, Bhatnagar RK (2002). Silencing of midgut aminopeptidase-N of *Spodoptera litura* by double-stranded RNA establishes its role as *Bacillus thuringiensis* toxin receptor.. J Biol Chem.

[19] Siqueira HAA, Gonzalez-Cabrera J, Ferre J, Flannagan R, Siegfried BD (2006). Analyses of Cry1Ab binding in resistant and susceptible strains of the European corn borer, *Ostrinia nubilalis* (Hubner) (Lepidoptera: Crambidae).. Appl Environ Microbiol.

[20] Siqueira HAA, Moellenbeck D, Spencer T, Siegfried BD (2004). Cross-resistance of CrylAb-selected *Ostrinia nubilalis* (Lepidoptera: Crambidae) to *Bacillus thuringiensis* delta-endotoxins.. J Econ Entomol.

[21] Bolin PC, Hutchison WD, Andow DA (1999). Long-term selection for resistance to *Bacillus thuringiensis* Cry1Ac endotoxin in a Minnesota population of European corn borer (Lepidoptera: Crambidae).. J Econ Entomol.

[22] Chaufaux J, Seguin M, Swanson JJ, Bourguet D, Siegfried BD (2001). Chronic exposure of the European corn borer (Lepidoptera: Crambidae) to CrylAb *Bacillus thuringiensis* toxin.. J Econ Entomol.

[23] Li HR, Oppert B, Higgins RA, Huang FN, Zhu KY (2004). Comparative analysis of proteinase activities of *Bacillus thuringiensis*-resistant and -susceptible *Ostrinia nubilalis* (Lepidoptera: Crambidae).. Insect Biochem Mol Biol.

[24] Li H, Oppert B, Higgins RA, Huang F, Buschman LL (2005). Susceptibility of Dipel-resistant and -susceptible *Ostrinia nubilalis* (Lepidoptera: Crambidae) to individual *Bacillus thuringiensis* protoxins.. J Econ Entomol.

[25] Siqueira HAA, Nickerson KW, Moellenbeck D, Siegfried BD (2004). Activity of gut proteinases from Cry1Ab-selected colonies of the European corn borer, *Ostrinia nubilalis* (Lepidoptera: Crambidae).. Pest Manag Sci.

[26] Khajuria C, Zhu YC, Chen M-S, Buschman LL, Higgins RA (2009). Expressed sequence tags from larval gut of the European corn borer (*Ostrinia nubilalis*): Exploring the genes potentially involved in *Bacillus thuringiensis* toxicity and resistance.. BMC Genomics.

[27] Coates BS, Sumerford DV, Lewis LC (2008). Mining an *Ostrinia nubilalis* midgut expressed sequence tag (EST) library for candidate genes and single nucleotide polymorphisms (SNPs).. Insect Molec Biol.

[28] Heckel DG (1994). The complex genetic basis of resistance to *Bacillus thuringiensis* toxin in insects.. Biocontrol Sci Tech.

[29] Oppert B, Kramer KJ, Beeman RW, Johnson D, McGaughey WH (1997). Proteinase-mediated insect resistance to *Bacillus thuringiensis* toxins.. J Biol Chem.

[30] Ferré J, Van RJ (2002). Biochemistry and genetics of insect resistance to *Bacillus thuringiensis.*. Annu Rev Entomol.

[31] Keller M, Sneh B, Strizhov N, Prudovsky E, Regev A (1996). Digestion of delta-endotoxin by gut proteases may explain reduced sensitivity of advanced instar larvae of *Spodoptera littoralis* to CryIC.. Insect Biochem Mol Biol.

[32] Coates BS, Sumerford DV, Lewis LC (2008). Segregation of European corn borer, *Ostrinia nubilalis,* aminopeptidase 1, cadherin, and bre5-like alleles, from a colony resistant to *Bacillus thuringiensis* Cry1Ab toxins, are not associated with F2 larval weights when fed a diet containing Cry1Ab.. J Insect Sci.

[33] Crespo ALB, Spencer T, Alves AP, Hellmich RL, Blankenship EE (2009). On-plant survival and inheritance of resistance to Cry1Ab toxin from *Bacillus thuringiensis* in a field-derived strain of European corn borer, *Ostrinia nubilalis*.. Pest Manag Sci.

[34] Alves AP, Spencer TA, Tabashnik BE, Siegfried BD (2006). Inheritance of resistance to the Cry1Ab *Bacillus thuringiensis* toxin in *Ostrinia nubilalis* (Lepidoptera: Crambidae).. J Econ Entomol.

[35] Crava CM, Bel Y, Lee SF, Manachini B, Heckel DG (2010). Study of the aminopeptidase N gene family in the lepidopterans *Ostrinia nubilalis* (Hübner) and *Bombyx mori* (L.): Sequences, mapping and expression.. Insect Biochem Mol Biol.

[36] Wang P, Zhang X, Zhang J (2005). Molecular characterization of four midgut aminopeptidase N isozymes from the cabbage looper, *Trichoplusia ni.*. Insect Biochem Mol Biol.

[37] Angelucci C, Barrett-Wilt GA, Hunt DF, Akhurst RJ, East PD (2008). Diversity of aminopeptidases, derived from four lepidopteran gene duplications, and polycalins expressed in the midgut of *Helicoverpa armigera*: identification of proteins binding the ä-endotoxin, Cry1Ac of *Bacillus thuringiensis*.. Insect Biochem Mol Biol.

[38] Budatha M, Meur G, Dutta-Gupta A (2007). A novel aminopeptidase in the fat body of the moth Achaea janata as a receptor for *Bacillus thuringiensis* cry toxins and its comparison with midgut aminopeptidase.. Biochem J.

[39] Budatha M, Meur G, Kirti PB, Dutta-Gupta A (2007). Characterization of *Bacillus thuringiensis* Cry toxin binding novel GPI anchored aminopeptidase from fat body of the moth *Spodoptera litura*.. Biotech Lett.

[40] Hua G, Tsukamoto K, Ikezawa H (1998). Cloning and sequence analysis of the aminopeptidase N isozyme (APN2) from *Bombyx mori* midgut.. Comp Biochem Physiol B Biochem Mol Biol.

[41] Bozic N, Vujcic Z, Nenadovic V, Ivanovic J (2003). Partial purification and characterization of midgut leucyl aminopeptidase of *Morimus funereus* (Coleoptera: Cerambycidae) larvae.. Comp Biochem Physiol B Biochem Mol Biol.

[42] Emmerling M, Chandler D, Sandeman M (2001). Molecular cloning of three cDNAs encoding aminopeptidases from the midgut of *Helicoverpa punctigera*, the Australian native budworm.. Insect Biochem Mol Biol.

[43] Kulkarni GV, Deobagkar DD (2002). A cytosolic form of aminopeptidase P from *Drosophila melanogaster*: molecular cloning and characterization.. J Biochem.

[44] Xu X, Yu L, Wu Y (2005). Disruption of a cadherin gene associated with resistance to Cry1Ac delta-endotoxin of *Bacillus thuringiensis* in *Helicoverpa armigera*.. Appl Environ Microbiol.

[45] Gahan LJ, Pauchet Y, Vogel H, Heckel DG (2010). An ABC Transporter Mutation Is Correlated with Insect Resistance to *Bacillus thuringiensis* Cry1Ac Toxin.. PLoS Genet.

[46] Ryan JW, Berryer P, Chung AY, Sheffy DH (1994). Characterization of rat pulmonary vascular aminopeptidase P in vivo: Role in the inactivation of bradykinin.. J Pharmacol Exp Ther.

[47] Cunningham DF, O'Connor B (1997). Proline specific peptidases.. Biochim Biophys Acta.

[48] Turner AJ, Hyde RJ, Lim J, Hooper NM (1997). Structural studies of aminopeptidase P: A novel cellular peptidase.. Adv Exp Med Biol.

[49] Yaron A, Mlynar D (1968). Aminopeptidase-P.. Biochem Biophys Res Commun.

[50] Siegfried BD, Spencer T, Crespo ALB, Storer NP, Head GP (2007). Ten years of monitoring for Bt resistance in the European corn borer: What we know, what we don't know and what we can do better.. Amer Entomol.

[51] Marçon PCRG, Young LJ, Steffey KL, Siegfried BD (1999). Baseline susceptibility of European corn borer (Lepidoptera: Crambidae) to *Bacillus thuringiensis* toxins.. J Econ Entomol.

[52] Bendtsen JD, Nielsen H, Heijne GV, Brunak S (2004). Improved prediction of signal peptides: SignalP 30.. J Mol Biol.

[53] Larkin MA, Blackshields G, Brown NP, Chenna R, McGettigan PA (2007). ClustalW and ClustalX version 20.. Bioinformatics.

[54] Pierleoni A, Martelli PL, Casadio R (2008). PredGPI: a GPI anchor predictor.. BMC Bioinformatics.

[55] Julenius K, Molgaard A, Gupta R, Brunak S (2005). Prediction, conservation analysis, and structural characterization of mammalian mucin-type *O*-glycosylation sites.. Glycobiol.

[56] Schultz FM, Bork P, Ponting CP (1998). A simple modular architecture research tool: Identification of signaling domains.. Proc Natl Acad Sci.

[57] Livak KJ, Schmittgen TD (2001). Analysis of relative gene expression data using real-time quantitative PCR and the 2[-Delta Delta C(T)] Method.. Methods.

[58] Huarong L, Oppert B, Higgins RA, Huang F, Buschman LL (2005). Characterization of cDNAs encoding three trypsin-like proteinases and mRNA quantitative analysis in Bt-resistant and -susceptible strains of *Ostrinia nubilalis*.. Insect Biochem Mol Biol.

[59] Khajuria C, Buschman LL, Chen MS, Muthukrishnan S, Zhu KY (2010). A gut-specific chitinase gene essential for regulation of chitin content of peritrophic matrix and growth of *Ostrinia nubilalis* larvae.. Insect Biochem Mol Biol.

[60] Khajuria C, Buschman LL, Chen MS, Zhu KY (2011). Characterization of six antibacterial response genes from the European corn borer (*Ostrinia nubilalis*) larval gut and their expression in response to bacterial challenge.. J Insect Physio.

[61] Pfaffl MW, Horgan GW, Dempfle L (2002). Relative expression software tool (REST) for group-wise comparison and statistical analysis of relative expression results in real-time PCR.. Nucleic Acids Res.

