# Supplementary material for: Identification of a Novel Aminopeptidase P-Like Gene (OnAPP) Possibly Involved in Bt Toxicity and Resistance in a Major Corn Pest (Ostrinia nubilalis)
Source: PLoS One. 2011 Aug 24;6(8):e23983. doi: 10.1371/journal.pone.0023983 (PMC3161092; doi:10.1371/journal.pone.0023983)
Supplement: Figure S1 — Primer efficiency test by qPCR. Efficiencies of each pair of primers were calculated using four 5-fold serial dilutions (1∶1, 1∶5, 1∶25, and 1∶125) in triplicates. iCycler software (Bio-Rad Laboratories) was used to determine slope, efficiency, and correlation co-efficient for each primer pairs. Summarized data are provided in Table 2. Correlation coefficients for all the primers are >0.99. (PDF) [file pone.0023983.s001.pdf]

Figure S1

OnAPP_P1

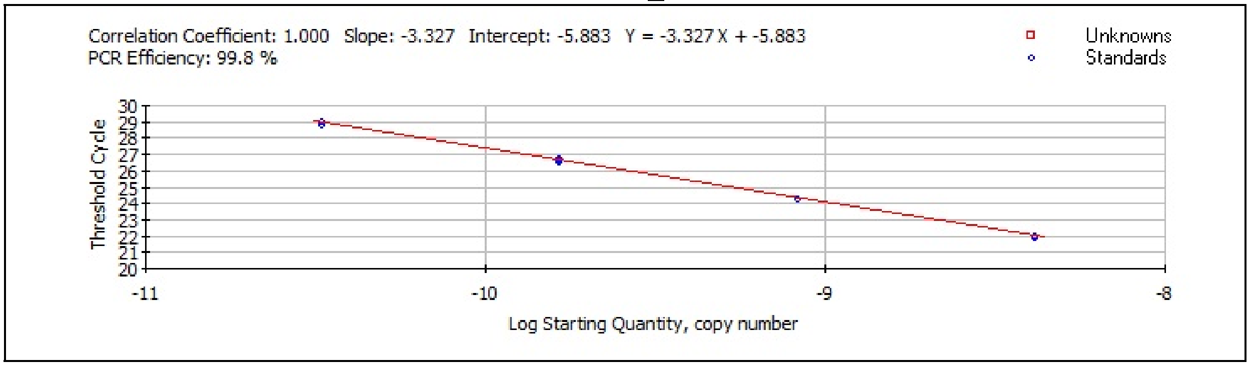

OnAPP_P2

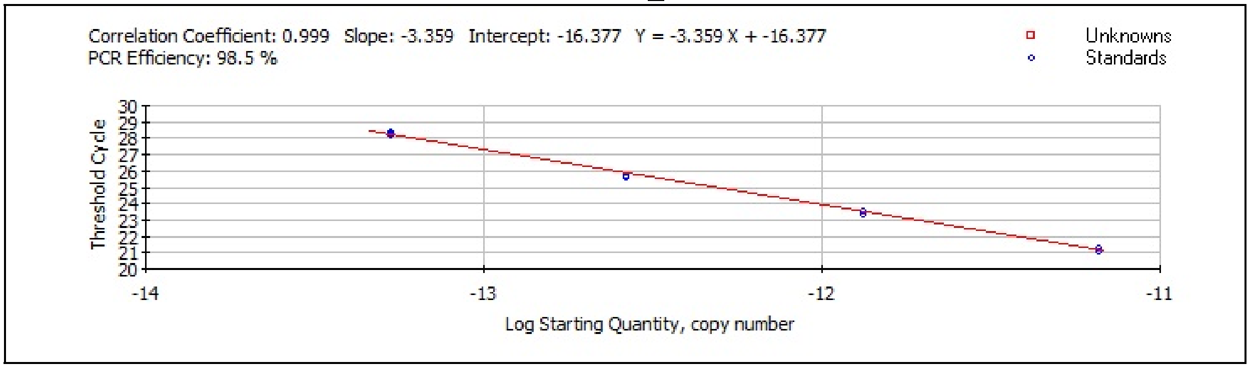

OnAPN1

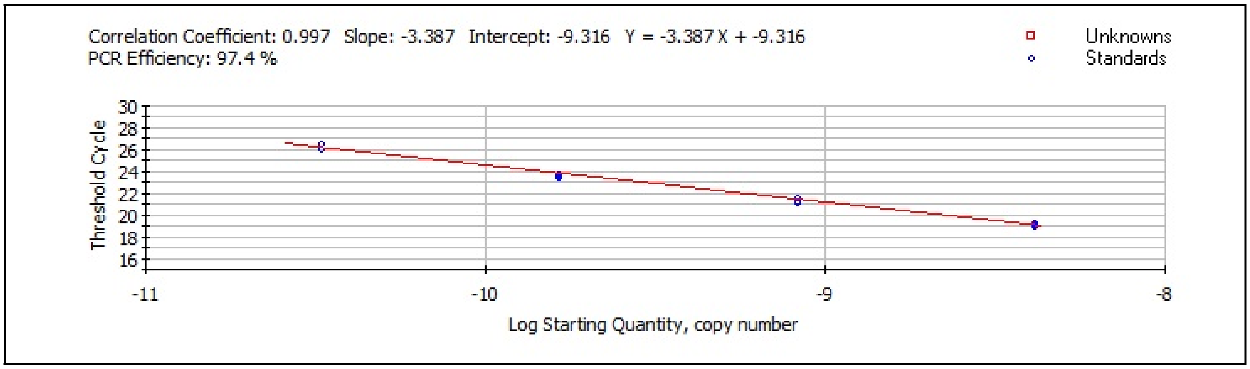

OnAPN2

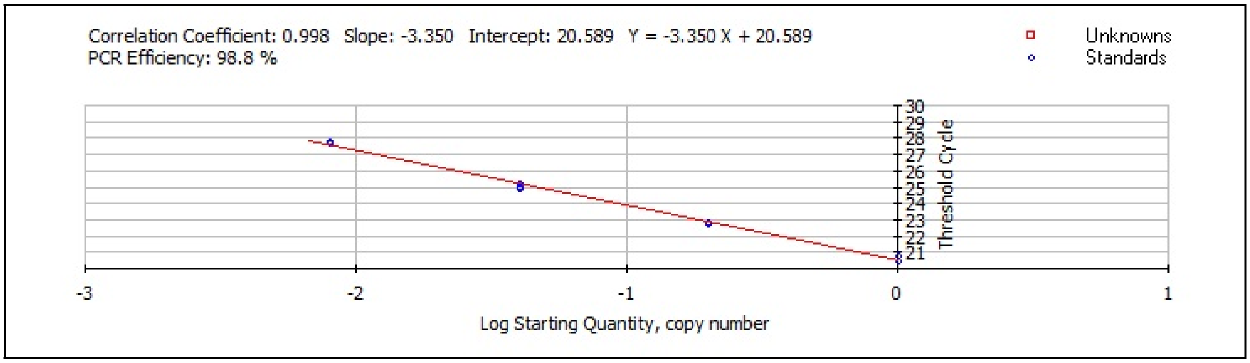

OnAPN3

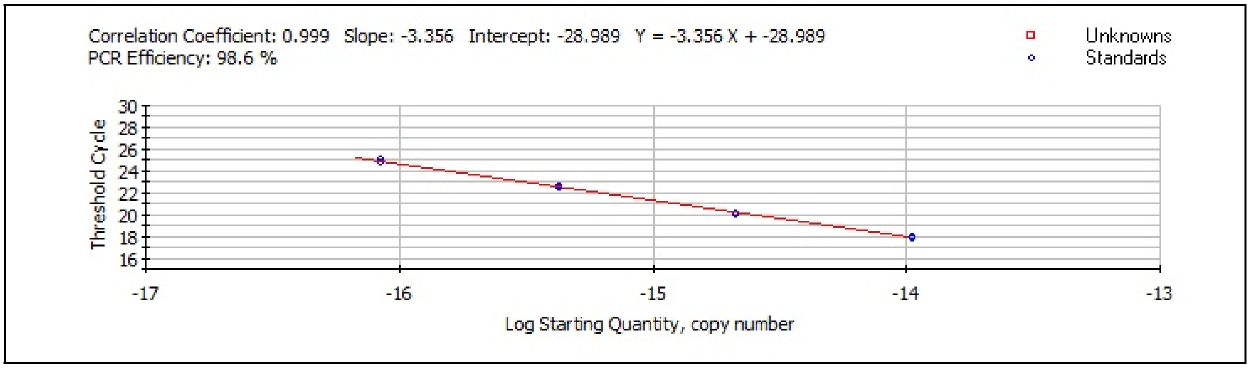

OnAPN4

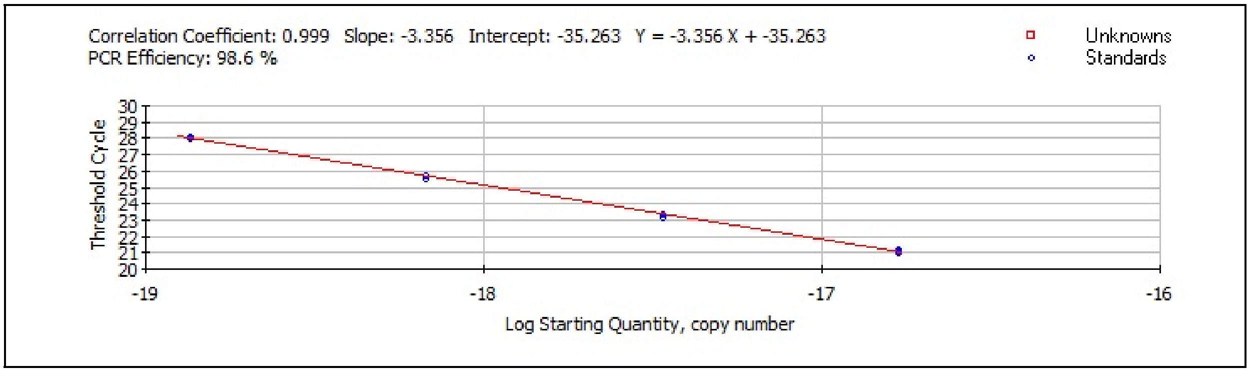

OnAPN5

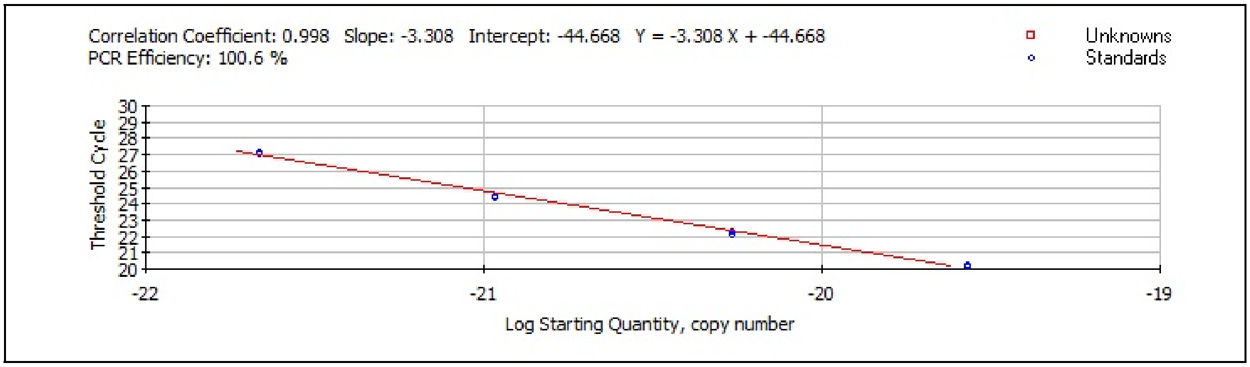

OnAPN6

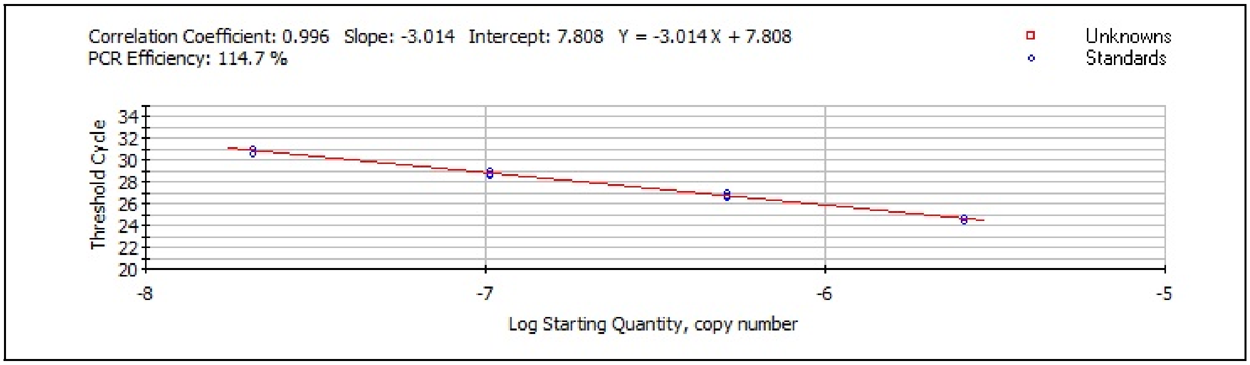

OnAPN7

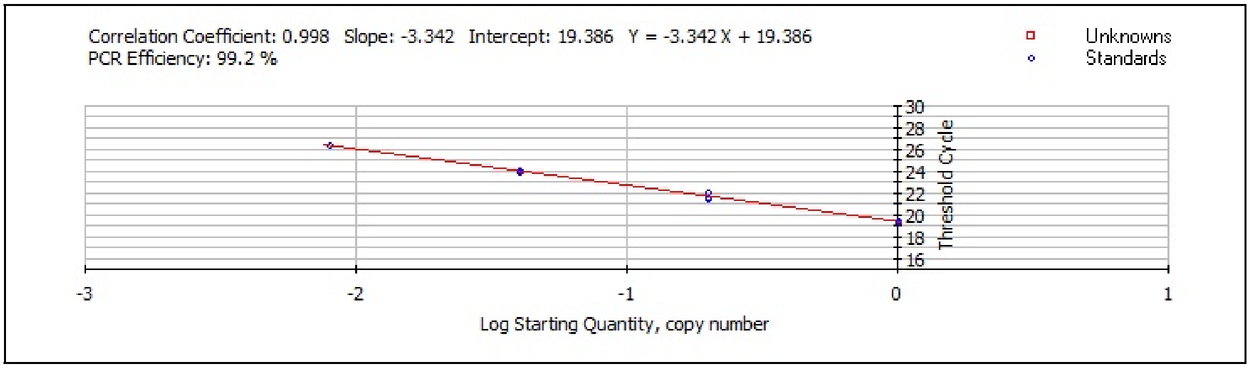

OnANP8

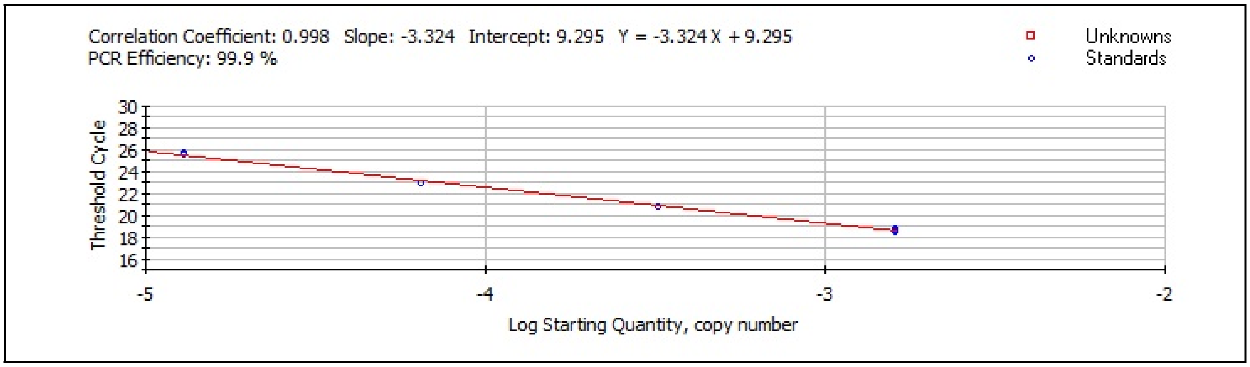

OnAPN9

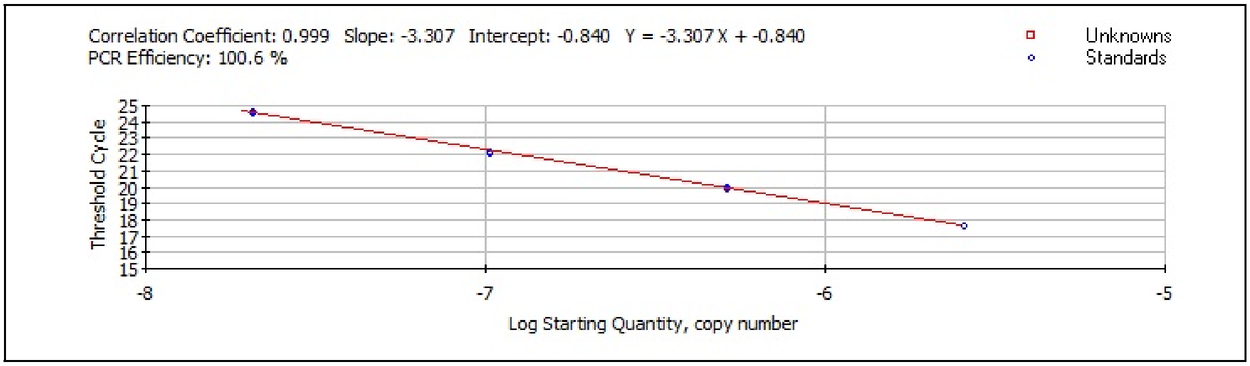
Correlation Coefficient: 0.999 Slope: -3.307 Intercept: -0.840 Y = -3.307 X + -0.840
PCR Efficiency: 100.6 %
Unknowns
Standards
Threshold Cycle
25
24
23
22
21
20
19
18
17
16
15
-8
-7
-6
-5
Log Starting Quantity, copy number

RPS3

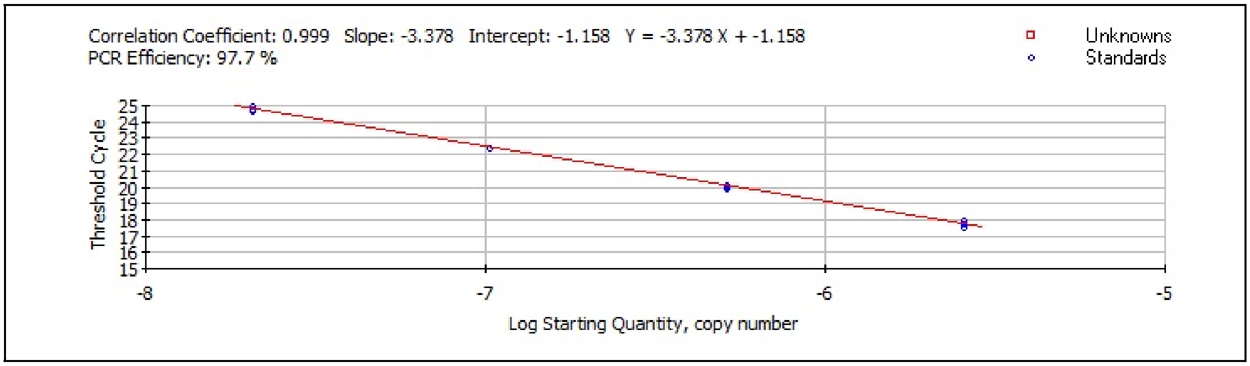
Correlation Coefficient: 0.999 Slope: -3.378 Intercept: -1.158 Y = -3.378 X + -1.158
PCR Efficiency: 97.7 %
Unknowns
Standards
Threshold Cycle
25
24
23
22
21
20
19
18
17
16
15
-8
-7
-6
-5
Log Starting Quantity, copy number
