# Supplementary figures and images for: Identification of a Novel Aminopeptidase P-Like Gene (OnAPP) Possibly Involved in Bt Toxicity and Resistance in a Major Corn Pest (Ostrinia nubilalis)
Source: PLoS One. 2011 Aug 24;6(8):e23983. doi: 10.1371/journal.pone.0023983 (PMC3161092; doi:10.1371/journal.pone.0023983)

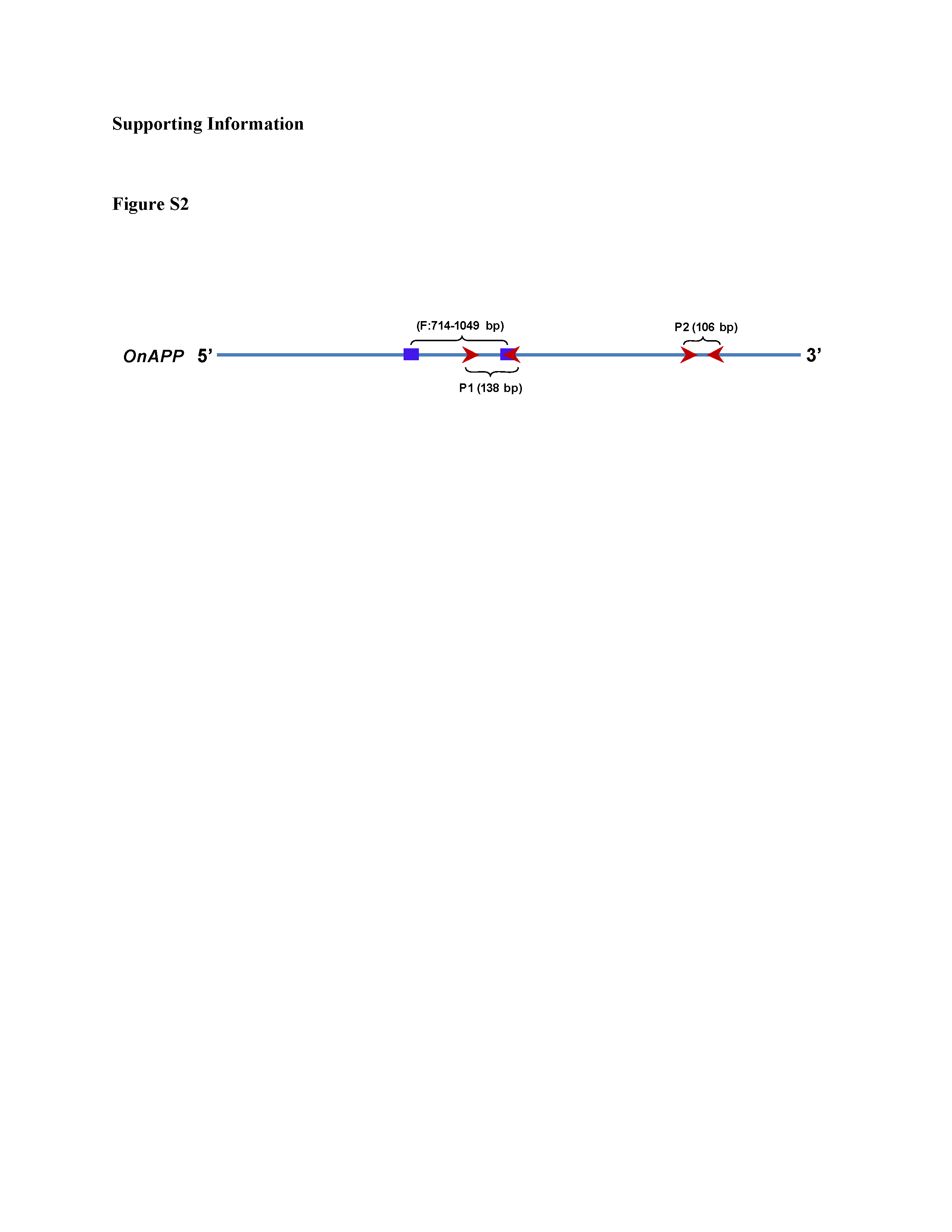

Supplement: Figure S2 — The design of the OnAPP dsRNA construction. The fragment (F) indicates the region used to generate the double stranded RNA for RNAi experiment. Only Cry1Ab-susceptible ECB larvae were used in this analysis. P1 and P2 indicated the location of primer set 1 and primer set 2 used to assess the expression of OnAPP in this study. (TIF) [file pone.0023983.s002.tif]
